# Interpretable Bayesian optimization for catalyst discovery

**DOI:** 10.1039/d5fd00159e

**Published:** 2026-01-27

**Authors:** Akhil S. Nair, Lucas Foppa, Matthias Scheffler

**Affiliations:** a The NOMAD Laboratory at the Fritz Haber Institute of the Max Planck Society Faradayweg 4-6 D-14195 Berlin Germany; b Institut für Chemie und Biochemie, Freie Universität Berlin Arnimallee 22 14195 Berlin Germany akhil.sugathan.nair@fu-berlin.de

## Abstract

Bayesian optimization (BO) efficiently explores vast design spaces using probabilistic surrogate models, enabling the guided discovery of materials with desired properties. However, most BO frameworks rely on the knowledge of a few key physical parameters (features) correlated with the materials property of interest. This is a challenge in heterogeneous catalysis, where the material properties are governed by an intricate interplay of multiple physical processes, and the mentioned parameters are typically unknown. Here, we introduce the Sparse Adaptive Representation-based Bayesian Optimization (SARBO) framework that utilizes the sure independence screening and sparsifying operator (SISSO) symbolic-regression method for on-the-fly selection of key physical parameters correlated with materials properties during BO. Crucially, SISSO takes into account nonlinear relationships and interactions between multiple parameters when selecting key features. We demonstrate that SARBO enables efficient navigation of the materials spaces and outperforms widely used feature-selection approaches for the simulated discovery of single- and dual-atom alloy surface sites capable of activating CO_2_, a critical step in the CO_2_ reduction reaction.

## Abstract 2

Symbolic regression guided adaptive feature selection improves the interpretability and performance of Bayesian optimization for catalyst discovery.

## Graphical abstract

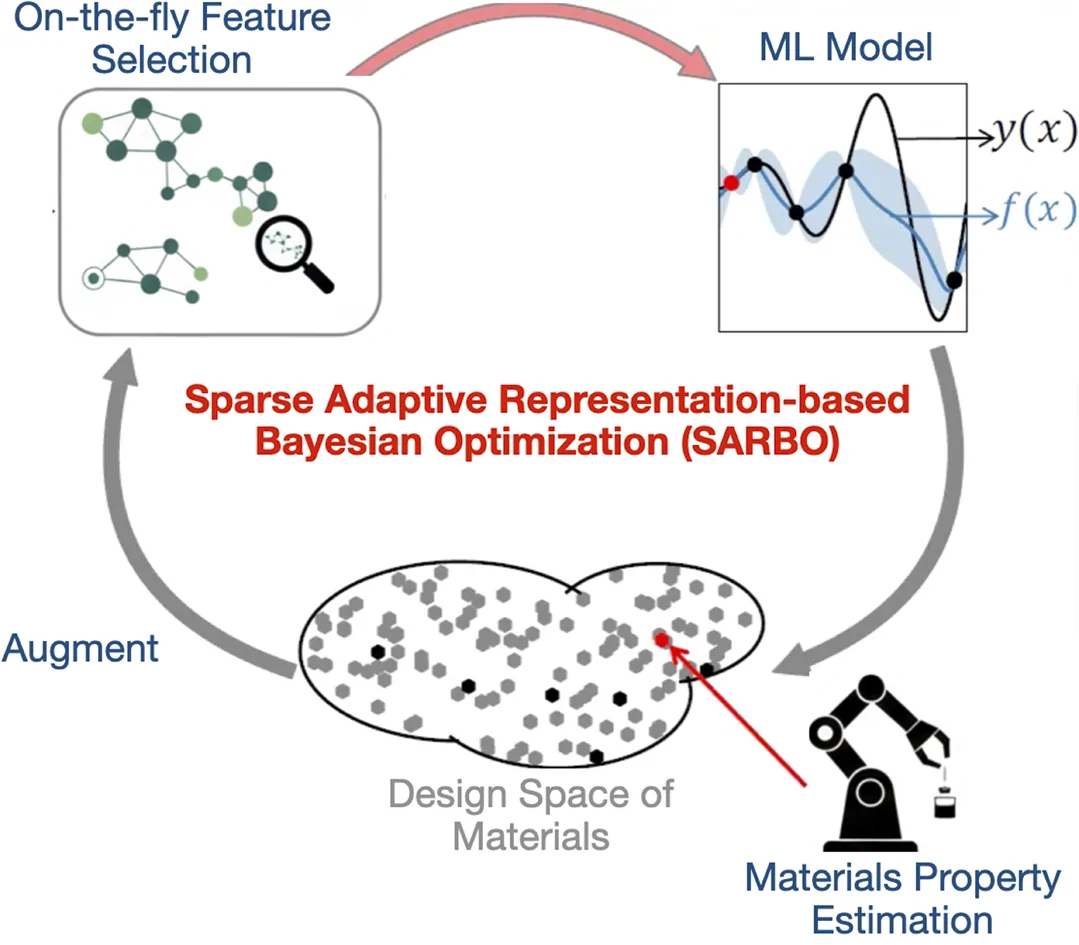

## 1 Introduction

The discovery of new materials is essential for progress in many technologies, including catalysis and energy storage.^1,2^ However, the population of possible materials or materials space, is practically infinite. Hence, the direct screening of materials *via* high-throughput experimentation or computational simulations is inefficient. This has motivated the development of autonomous materials discovery platforms, where closed-loop systems selectively acquire the next materials to be investigated and incorporate the feedback from experiments or simulations to improve the efficiency of materials discovery.^3,4^ Bayesian optimization (BO) has been an essential component of such platforms, as it is well-suited for optimizing objective functions that link physical parameters describing the material properties, termed features, to a certain target materials property.^5,6^ In the context of materials discovery, the optimization could refer to identifying materials with optimal properties, *e.g.*, with high or low values. BO has two main components: a surrogate model and an acquisition function.^7^ The surrogate (machine-learning) model approximates the objective function, mapping the features to the material properties based on the observed data. Gaussian Process (GP) and Random Forests (RF) are the most common surrogate models for BO due to their probabilistic formulation^8^ and robustness to noise,^9^ respectively. GP models the objective function non-parametrically as a distribution over smooth functions, enabling both accurate predictions and principled uncertainty estimates. RF uses an ensemble of decision trees, providing strong predictive performance while offering practical uncertainty estimates through ensemble variance. The acquisition function uses predictions and uncertainty estimates from the surrogate model to suggest the next materials from the design space, *i.e.* the set of all materials represented through a set of features, available for evaluation. The selected materials are then evaluated, added to the training data, and the surrogate model is updated, completing one BO iteration. Thus, BO balances the selection of materials with predicted desired properties, *i.e.*, the model exploitation, and the exploration of portions of the design space that may not be well represented in the training set. By focusing on such carefully selected samples, BO significantly reduces the effort, *e.g.*, the cost for evaluation of the materials property, necessary to identify materials with desired properties.^10,11^

Despite its successes in materials discovery applications,^12–14^ the efficiency of BO decreases as the number of features increases. This is because the search domain increases exponentially with the number of features, making it difficult to identify optimal regions within an affordable number of property evaluations.^15^ This is a limitation in fields such as heterogeneous catalysis, where the catalytic performance of a material is governed by an intricate interplay of multiple physical processes, and the key features correlated with the catalyst performance are often unknown. In such cases, BO might utilize significant resources to explore irrelevant portions of the design space. In order to mitigate this “curse of dimensionality” challenge in BO, several methods have been proposed where the surrogate model parameters can be tuned to focus on the features that matter most, ignoring irrelevant ones.^16,17^ However, such methods (*e.g.*, automatic relevance determination^16^) are typically tied to a specific surrogate model (*e.g.*, GP) and therefore are not easily transferable to other model classes. Feature-selection (FS) methods, in turn, automatically identify a few relevant features, out of many offered, potentially relevant features. Then, the surrogate model uses only these most relevant features during BO.^18^ However, most commonly used FS methods rely on linear correlation metrics and consequently fail to capture complex, non-linear feature interactions. Therefore, they may neglect features that appear redundant or weakly correlated with the target property when considered individually, but that might become relevant when they are combined with other features. In addition, the FS is typically performed based on the initial data set and this fixed set of features is utilized throughout the BO iterations. This might be an issue in materials science applications, since the relevance of features can change as new materials are acquired and included in the training data set. Indeed, the initial training set might not be representative of the entire design space. Thus, BO with a fixed feature set may overlook relevant materials. To address this issue, Rajabi-Kochi *et al.* introduced adaptive FS within each BO iteration, demonstrating improved performance over traditional BO approaches.^19^ However, this framework did not account for the non-linear interactions between features. As a result, even with adaptive representations, features which are potentially relevant to describe the target property might be excluded, causing FS methods to function as bad advisors, limiting the effectiveness of BO.

**Fig. 1:**
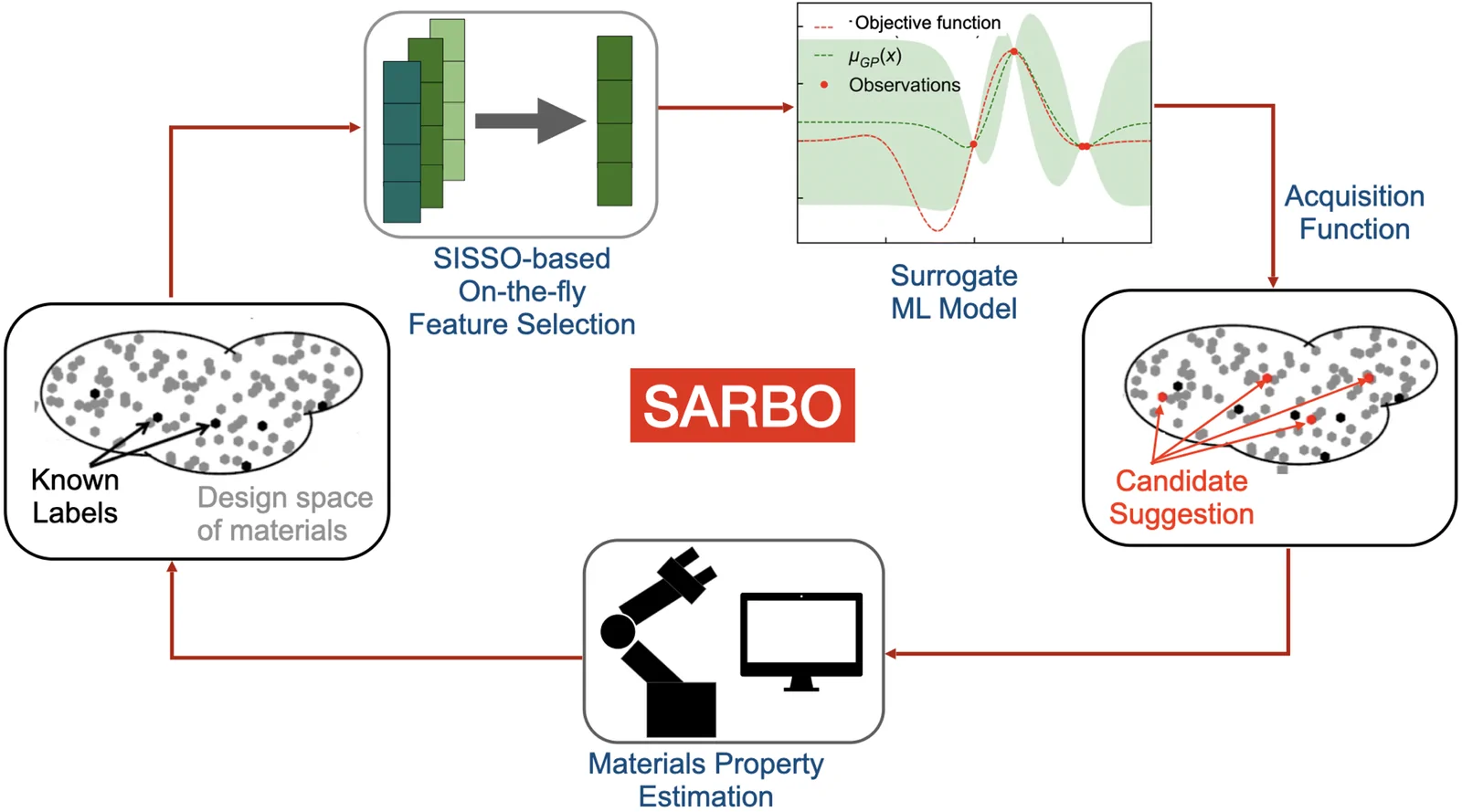
Schematic of the SARBO framework. The iterative cycle begins with known labelled data, proceeds through SISSO-based on-the-fly feature selection to adaptively represent materials, training a surrogate ML model, candidate suggestion *via* acquisition function and property estimation until exhausting a pre-defined budget or achieving a desired optimization goal.

In this work, we present the Sparse Adaptive Representation-based Bayesian Optimization (SARBO) framework to address the curse of dimensionality faced by standard BO approaches (Fig. 1). This framework utilizes the Sure-Independence Screening and Sparsifying Operator (SISSO) symbolic-regression method for on-the-fly selection of features, taking into account nonlinear target-feature relationships and interaction between features.^20^ SISSO identifies, at each iteration of the BO, a small set of physico-chemical features correlated with the target property, also termed as materials genes.^21^ Then, a surrogate model, *e.g.*, GP, RF or the SISSO model itself, is trained only with the features selected by SISSO. This strategy refines the design space description on-the-fly. In addition, SISSO is an inherently interpretable method in the sense that it provides models as analytical expressions or descriptors. These expressions make the most relevant correlations between the features and the target property explicitly. In the prior work,^22^ we have demonstrated how SISSO can be directly used as a surrogate model for BO, combining both its feature selection, predictive ability and uncertainty estimates by considering ensembles of SISSO models. In the current study, we repurpose SISSO as a general FS method that can be utilized with any surrogate model. In particular, SISSO-based FS can be combined with GP, taking advantage of the principled uncertainty estimates provided by this approach. We critically evaluate the performance of the SARBO approach against standard BO integrating alternative FS strategies, demonstrating its superior performance in identifying the surface sites of single- and dual-atom alloys for CO_2_ activation, a critical step in CO_2_ reduction reaction.

## 2 Methods

### 2.1 Dataset

**Fig. 2:**
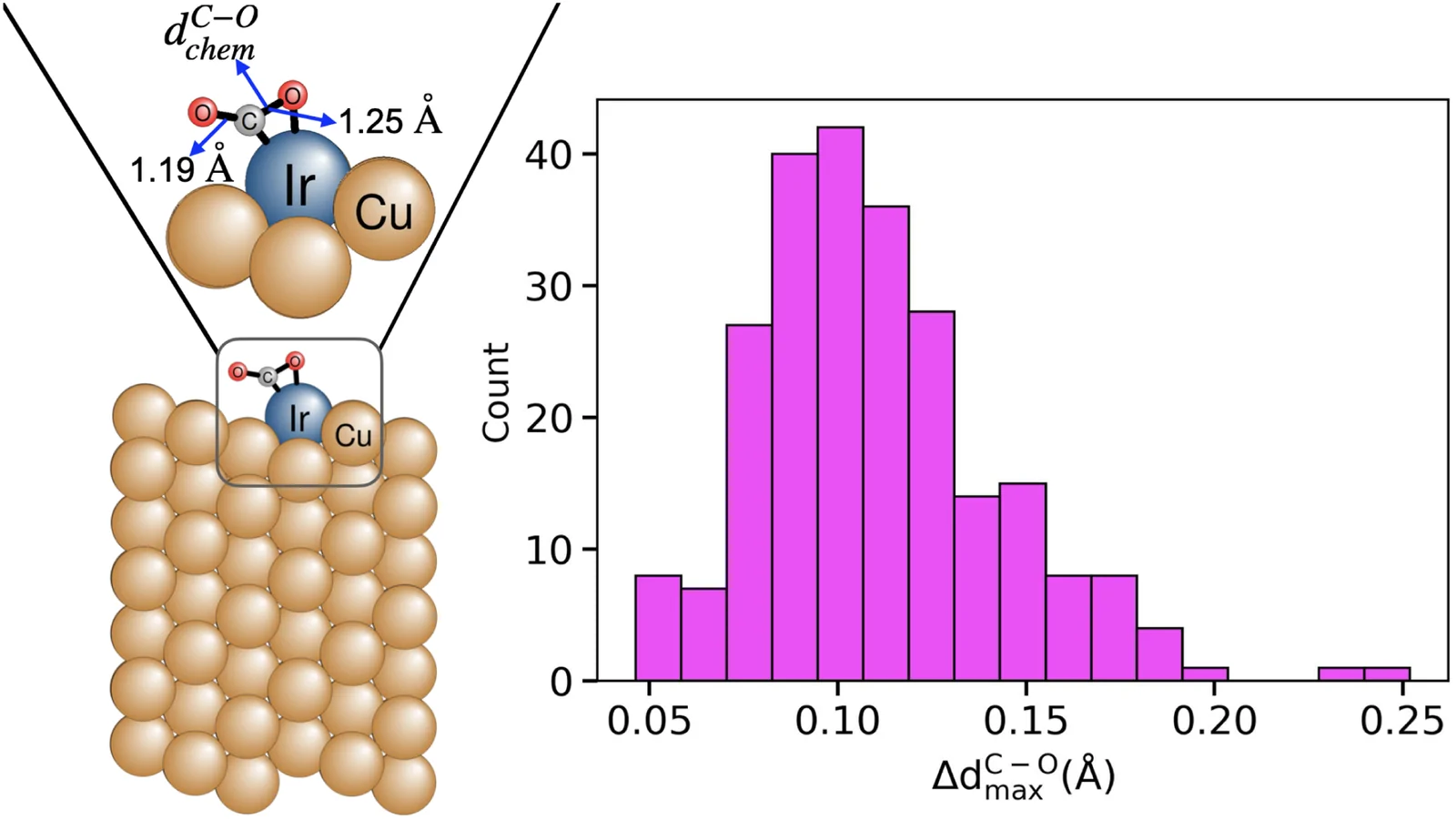
(left) Representative atomic structure of the Ir@Cu(211) SAA surface. The local coordination environment of the single-atom is shown in an enlarged view. The target property Δ*d*^C–O^_max_ is evaluated as the difference between the largest distance between C and O atoms in chemisorbed CO_2_ (indicated by *d*^C–O^_chem_) and the C–O bond length in the gas phase CO_2_ molecule, (right) distribution of Δ*d*^C–O^_max_ values across the dataset of 240 surface sites in the considered dataset.

The dataset considered in this study consists of 218 surface sites of single-atom alloys (SAAs) and 16 surface sites of dual-atom alloys (DAAs).^23^ In these materials, one (two) isolated atom(s) of a metal, termed dopant(s), is (are) dispersed and stabilized on a different metal, termed host^24–30^ (Fig. 2). SAAs and DAAs display unique electronic properties compared to monometallic systems or stoichiometric alloys^31–34^ that can be used to design new catalysts. The SAAs and DAAs considered in this work were constructed using surface terminations (111), (100), (110), (211) and (0001) of Cu, Pd and Zn hosts with dopants corresponding to elements of periodic table groups 8–13. In addition, 6 monometallic sites of Pd(211) surface is also included in the dataset. The CO_2_ chemisorption was evaluated for each of the surface sites using density functional theory (DFT) calculations with the mBEEF^35^ meta-generalized gradient approximation for electron exchange and correlation as implemented in the FHI-aims code.^36,37^ mBEEF has been shown to provide an accurate description of CO_2_ binding on SAA surfaces by previous benchmarking studies.^38,39^ The maximum C–O bond elongation in chemisorbed CO_2_, denoted as Δ*d*^C–O^_max_, is taken as the target property. It is estimated as;

1 Δ*d*^C–O^_max_ = *d*^C–O^_chem_ − *d*^C–O^_eq_

where *d*^C–O^_chem_ is the largest distance between C and O atoms in the chemisorbed CO_2_ molecule, and *d*^C–O^_eq_ is the C–O bond distance in the gas-phase CO_2_ molecule. This bond elongation measures the ability of the surface site to activate CO_2_, a prerequisite for subsequent steps in CO_2_ reduction. The larger Δ*d*^C–O^_max_, weaker is the C–O bond and the more likely it is that the molecule can be converted.^40,41^ Thus, the BO approach in this work aims at finding surface sites of SAAs and DAAs that maximize Δ*d*^C–O^_max_.

The 26 offered features can be classified in five different types: elemental properties of the (i) host and (ii) dopant such as d-orbital radius and electronegativity, (iii) properties related to the CO_2_ adsorption such as its adsorption energy, (iv) elemental and geometrical properties of the surface sites such as averaged d-orbital radius and coordination number, and (v) properties of the surface sites plus nearest neighbours such as generalized coordination number^42^ (see Table S1 for the full list of features). It should be noted that adsorption-related properties are included as features to model Δ*d*^C–O^_max_ because they are required to achieve accurate SISSO models. For example, including these features increased the training performance from an *R*^2^ of 0.4 to 0.74. This approach, however, presupposes knowledge of adsorption properties that are only available after performing DFT calculations. This reliance could, in principle, be alleviated by increasing the model complexity or by adopting the hi-SISSO strategy,^43^ although we find that the latter only slightly improve the performance of the model in this case (see Fig. S1). Because our focus is on evaluating optimization performance using a dataset in which these properties are available for all surface sites rather than predicting Δ*d*^C–O^_max_ for previously unseen surface sites, we did not explore further alternatives to circumvent the use of the adsorption-related properties. For the case of DAAs, we have only considered bridge sites between the two dopant atoms for CO_2_ adsorption and hence the elemental properties of the dopant are taken as the average of the properties of the two atoms. This dataset is particularly suitable for our study as Δ*d*^C–O^_max_ could be governed by multiple factors of various origins, such as dopant and host chemistry, surface structure and local coordination. Thus, the different SAAs and DAAs may require descriptions based on different features, making this a useful test case for adaptive feature selection.

### 2.2 Sparse adaptive representation-based Bayesian optimization (SARBO)

The SARBO framework is designed for efficient and interpretable materials discovery in high-dimensional feature spaces. BO operates on two different pools of materials: (i) an initial labeled pool of (few) materials 
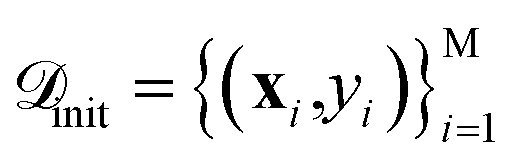
 for which the target property is known and (ii) a large pool of unlabelled candidate materials 
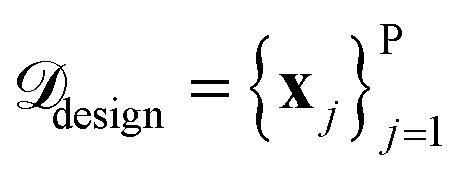
, for which the target property is not known. Each material **x** is represented by a set of initially offered features ***ϕ***_total_.

The closed-loop SARBO proceeds as follows:

(1) Descriptor learning with SISSO: the core component of SARBO utilizes SISSO for adaptive feature selection. During each BO iteration, SISSO processes the current 
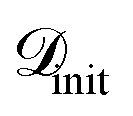
 to construct an extensive space of candidate features ***Φ*** by applying a set of mathematical operations to ***ϕ***_total_. These operations are unary (log, exponential, square) and binary (addition, subtraction, multiplication). Thus, they capture nonlinearities and interactions between features. Then, the sure-independence screening procedure followed by 
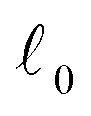
-regularized regression is applied to select a model of the form:
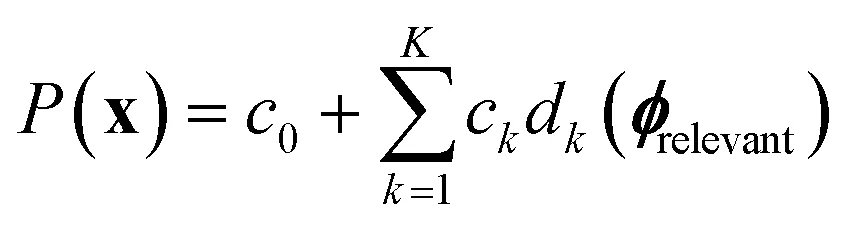
where {*d*_*k*_} represent the selected expressions. These expressions are components of a descriptor vector, and they depend on few, relevant features ***ϕ***_relevant_, from all initially offered features. *K* is the descriptor dimensionality and *c*_*k*_ are fitted coefficients. We note that even though the model above is linear, the nonlinearities are included in the expressions generated by SISSO. Also, the term “features” in this work refers to what is called “primary features” in the SISSO approach.^44^ The immense space of “SISSO-created features” (billions), *i.e.* candidate descriptors, only plays a hidden role in the current work.

(2) Adaptive representation: the SISSO model is used to redefine the design space representation for subsequent BO iterations. The materials are represented using only the set of features ***ϕ***_relevant_ ⊂ ***ϕ***_total_ that appear in the SISSO model. This creates a minimal, relevant subset of the original set of features.

(3) Surrogate modelling: a surrogate model *f*(**x**) is trained on 
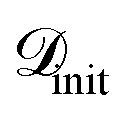
 represented only by ***ϕ***_relevant_ to approximate the objective function. The model provides a mean prediction *µ*(**x**) and an uncertainty estimate *σ*(**x**) for all 
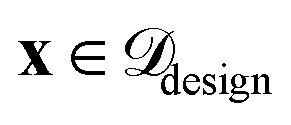
. This work demonstrates SARBO using GP and RF as surrogates where *σ*(**x**) is estimated as the standard deviation of the posterior distribution and predictions across ensemble trees, respectively.

(4) Acquisition and evaluation: we employ expected improvement (EI)^45^ and upper confidence bound (UCB)^46^ as acquisition functions as they balance exploitation and exploration for BO. For both acquisition functions, the next candidate(s) are selected by maximizing:
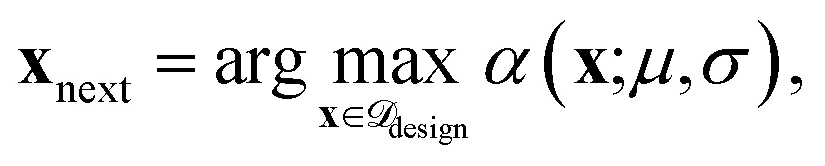
where *α*(**x**) represents the either EI or UCB scores. The target property *y*_next_ for **x**_next_ is evaluated, and the dataset is updated: 
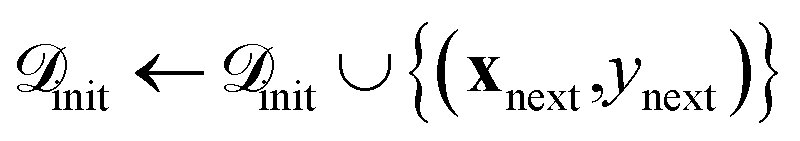
.

## 3 Results and discussions

### 3.1 Comparison of feature selection performance

We first compared the performance of SISSO-based feature selection (FS) with three other FS methods: recursive feature elimination (RFE),^47^ mutual information (MI),^48^ and minimum redundancy maximum relevance (MRMR).^49^ RFE iteratively prunes features based on their importance scores obtained from a RF model. MI retains features with the highest mutual information with the target property. Finally, MRMR selects features that maximize relevance to the target property while minimizing redundancy among them. RFE and MI are performed with 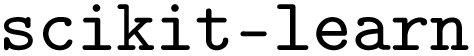.^50^ MRMR is performed with the 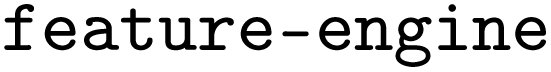 package.^51^ We trained GP and RF models for the target Δ*d*^C–O^_max_ by splitting the dataset into 80% train and 20% test data. The test-set performance of the models obtained with the features selected by the methods SISSO, RFE, MI, and MRMR is shown in Table 1 along with a baseline (labelled NoFS) corresponding to using the entire set of features.

**Table 1:** Performance comparison of feature selection (FS) methods using Gaussian Process (GP), Random Forests (RF), and SISSO. Considered FS methods include recursive feature elimination (RFE), mutual information (MI), minimum redundancy maximum relevance (MRMR), and SISSO-based feature selection. Metrics reported are the coefficient of determination (*R*^2^), mean absolute error (MAE), and root mean squared error (RMSE). For each method, absolute values are shown, and values in parentheses indicate ratios relative to the baseline (NoFS) using the complete set of features without any selection. For SISSO, the labels “SM” and “FS” indicate the scenarios when it is used directly as the surrogate model and only for feature selection, respectively

FS method	*R* ^2^			MAE (Å)			RMSE (Å)		
	GP	RF	SISSO-SM	GP	RF	SISSO-SM	GP	RF	SISSO-SM
NoFS	0.763	0.702	0.753	0.012	0.013	0.013	0.017	0.019	0.017
SISSO-FS	0.844 (1.106)	0.719 (1.024)	0.753 (1.0)	0.010 (0.835)	0.013 (0.970)	0.013 (1.0)	0.014 (0.812)	0.019 (0.971)	0.017 (1.0)
MI	0.644 (0.844)	0.646 (0.920)	0.695 (0.923)	0.017 (1.411)	0.015 (1.111)	0.015 (1.141)	0.021 (1.225)	0.021 (1.091)	0.019 (1.111)
RFE	0.650 (0.852)	0.655 (0.933)	0.648 (0.861)	0.016 (1.395)	0.015 (1.087)	0.017 (1.321)	0.021 (1.215)	0.021 (1.077)	0.021 (1.193)
MRMR	0.671 (0.880)	0.597 (0.850)	0.648 (0.861)	0.015 (1.269)	0.016 (1.157)	0.017 (1.321)	0.020 (1.177)	0.022 (1.164)	0.021 (1.193)

Let us first examine the performance of the GP models across the different FS methods. GP trained on SISSO selected features (labelled SISSO-FS) show the best performance among all methods, achieving the highest *R*^2^ (0.844) and the lowest MAE (0.01 Å) and RMSE (0.014 Å) values. This represents an improvement over the full-feature baseline. In contrast, the MI, RFE, and MRMR approaches all lead to reduced GP performance, with consistently higher errors. A similar trend is observed for the RF models: SISSO-based FS again provides the highest performance (*R*^2^ = 0.719, MAE = 0.013 Å, RMSE = 0.019 Å). However, the relative gains are more modest than for GP, reflecting the robustness of RF to irrelevant or redundant features *via* ensemble averaging. The remaining FS methods once again underperform relative to the full-feature baseline, exhibiting lower *R*^2^ and higher MAE, RMSE values. When SISSO is used as a surrogate model (labelled SISSO-SM) with its default FS, it is observed to have an intermediate performance between GP and RF. When combined with other FS methods, SISSO-SM generally performs better than RF for MI but is slightly worse than GP, and for RFE and MRMR, it tends to underperform compared to GP while being comparable to or slightly worse than RF. Table S2 shows the features selected by each FS method. While an overlap is observed for the selected features, only SISSO-FS uniquely captures effects associated with the host surface, single-atom dopant, surface site and the coordination environment, which are likely critical for describing CO_2_ activation. In contrast, the other FS methods systematically overlook at least one of these key factors. The enhanced performance observed with SISSO-FS compared to using the full set of features can be attributed to the reduction of redundancy and irrelevant information present in the full feature set. By relying on the most informative features, surrogate models can more effectively capture the underlying relationships, leading to improved prediction accuracy and generalization.

Overall, these results highlight SISSO as the most effective FS strategy among the considered methods, and GP as the surrogate model achieving the highest predictive performance, particularly when combined with SISSO-selected features. This suggests that even when using non-SISSO surrogate models such as GP or RF, employing SISSO solely for feature selection can provide substantial gains, as it identifies the most informative features while leveraging the flexibility of other surrogate models to learn complex feature-target relationships. Hence, in the following discussion, we focus on GP and RF surrogate models.

### 3.2 Performance of SARBO framework

We evaluate SARBO’s performance through after-the-fact simulated BO campaigns. After-the-fact means that the target property is not evaluated for a selected candidate surface site *via* DFT calculations, but it is taken from the complete dataset. Indeed, a small fraction (*f*_train_) of the total dataset is used as the initial data 
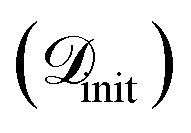
 for training, while the remaining data constitute a hidden candidate pool 
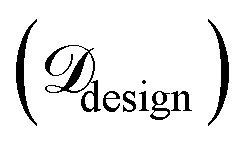
. This approach mimics real-world materials discovery scenarios where the optimal candidates are unknown *a priori* and has been adapted for comparing and benchmarking various BO methodologies in previous studies.^52–54^ The optimization is performed for *N*_iter_ iterations. At each iteration, the surrogate models 
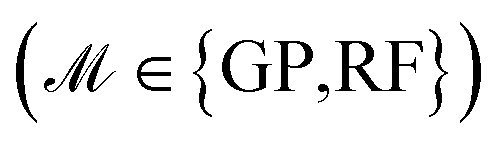
 are trained. They provide predictions for the targets Δ*d*^C–O^_max_, along with uncertainty estimates *σ*(**x**). The acquisition functions (*α* ∈ {EI, UCB}) then select a batch (*b*) of candidates **x**_next_ = arg max *α*(**x**), whose values of the target Δ*d*^C–O^_max_ are added to the training dataset. Finally, the FS method selects the relevant features for the next iteration. Since the optimization objective targets maximization of Δ*d*^C–O^_max_, we reserve a fraction (*f*_top_) of top candidates (highest Δ*d*^C–O^_max_ value) in 
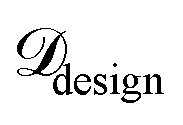
, excluding them from 
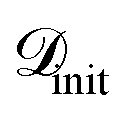
. We monitor the performance of the BO using two metrics: the highest observed Δ*d*^C–O^_max_ value at each iteration and the cumulative count of surface sites improving the target property, *i.e.*, with Δ*d*^C–O^_max_ values larger than the best known value in the previous iteration. Each BO campaign is performed *N*_trial_ times with different randomly selected initial training sets. Thus, confidence intervals are obtained for the BO campaigns. For all the results presented in the manuscript, we consider *f*_train_ = 0.1, *f*_top_ = 0.2, *b* = 5, *N*_iter_ = 40 and *N*_trial_ = 10, unless stated otherwise.

We begin by comparing the performance of SARBO against standard BO under two distinct scenarios. The first is a baseline where BO operates on the full feature set without any selection (NoFS). The second, labelled *Static*, employs a SISSO-derived feature set determined once at the outset and held fixed throughout all BO iterations. This static condition represents a data-rich ideal, where sufficient data exists *a priori* for feature selection, a scenario uncommon in typical BO experiments but plausible when leveraging pre-existing data from low-fidelity simulations or a closely related property.

**Fig. 3:**
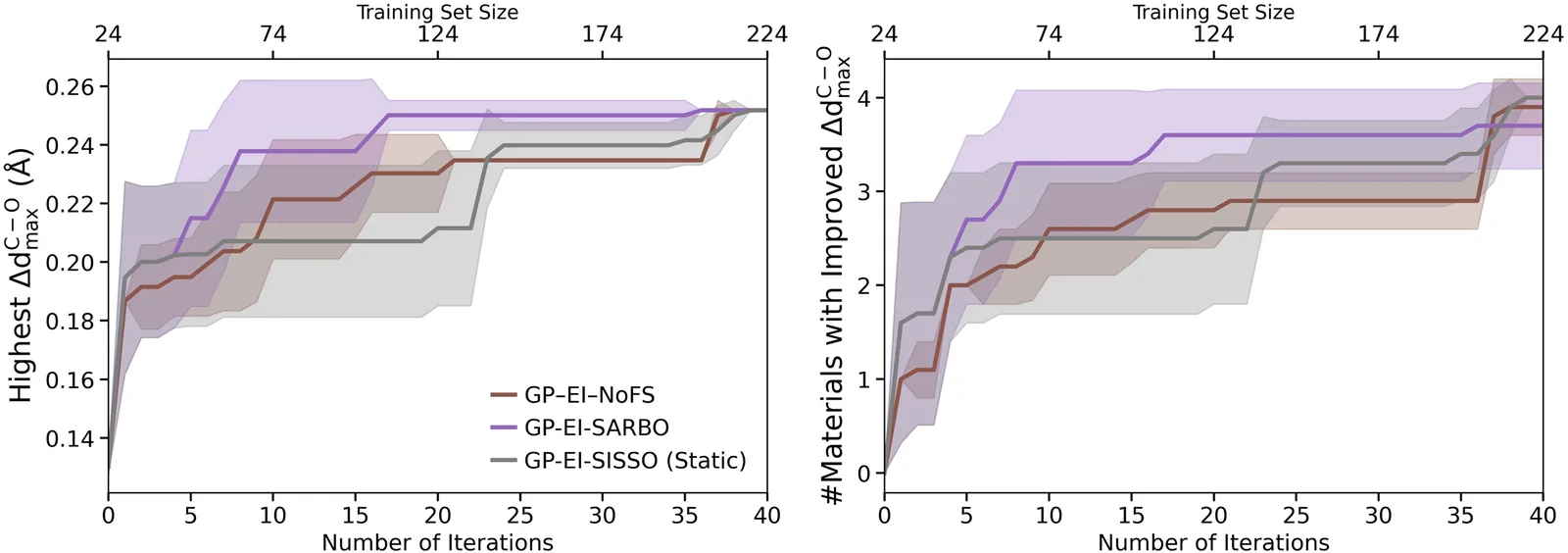
Performance comparison of SARBO against baseline Bayesian optimization (BO) methods: BO with no feature selection (NoFS) and BO with a static, one-time SISSO feature selection (SISSO-Static). (left) Evolution of the maximum Δ*d*^C–O^_max_ value over 40 iterations and (right) cumulative count of materials improving the target property. Training set sizes at selected iterations are shown on the top *x*-axis of both panels. Shaded regions indicate standard deviation across 10 independent trials.

Fig. 3 shows the results for the GP surrogate model and the EI acquisition function. The evolution of the highest Δ*d*^C–O^_max_ values identified by BO with the number of iterations is shown in the left panel. The highest Δ*d*^C–O^_max_ value around 0.25 Å is identified at *N*_iter_ = 17 by SARBO. Conversely, *N*_iter_ = 37 is needed for the standard BO approach utilizing the full set of features. While the static feature selection method shows a marginal performance improvement over the standard BO in the initial and final stages, it still required *N*_iter_ = 38 to achieve the highest target value. We also analyzed the evolution of the number of surface sites with improved Δ*d*^C–O^_max_ identified by BO with the number of iterations (Fig. 3, right panel). SARBO exhibits a small number of early, high-impact improvements and quickly reaches the vicinity of the global optimum, after which few further improvements are possible. In contrast, both standard and static feature set-based BO accumulate many incremental improvements over a larger number of iterations, reflecting slower progress through lower-quality regions of the design space. Overall, these results demonstrate that SARBO achieves substantially faster convergence toward the optimal region of the design space, indicating that the adaptively selected features from SISSO models provide a more informative and efficient representation of the design space for the surrogate model. Although an initial, one-time feature selection can improve performance, it is ultimately outperformed by SARBO. This static approach also carries the prerequisite of a substantial initial dataset for the feature selection itself. These results collectively underscore the critical advantage of SARBO’s adaptive feature adaptation.

**Fig. 4:**
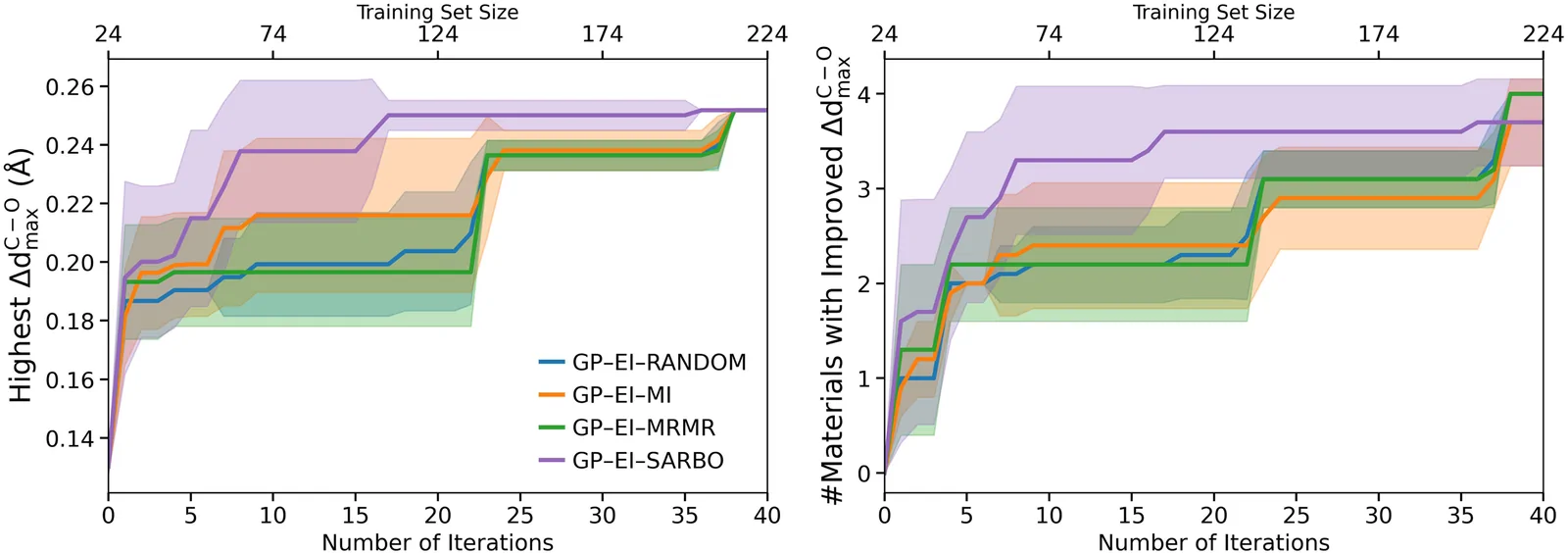
Performance comparison between BO with different feature selection strategies: (left) maximum Δ*d*^C–O^_max_ evolution, (right) cumulative improvements in target property. Shaded regions indicate standard deviation across 10 independent trials.

We now compare the performance of various FS methods when integrated into an adaptive BO loop like the one used in SARBO, for the case of the GP surrogate model with the EI acquisition function. Fig. 4 compares SARBO with BO approaches that employ MI and MRMR. Additionally, we also consider a random-selection baseline (RANDOM) where features are selected randomly without any measurement of feature importance. For consistency, all methods use the same number of features selected by SARBO. It can be seen that SARBO outperforms the other FS strategies in identifying surface sites with the highest Δ*d*^C–O^_max_. Indeed, SARBO reaches the maximum mean value around 0.25 Å by iteration *N*_iter_ = 17, whereas the other methods require *N*_iter_ = 38 to achieve the same performance. This advantage is already evident at early iterations: at *N*_iter_ = 10, the highest Δ*d*^C–O^_max_ values are 0.20 (RANDOM), 0.21 (MI), 0.19 (MRMR), and 0.24 Å (SARBO). A similar trend appears in the cumulative number of improved candidates. SARBO identifies improved candidates earlier and with larger mean values relative to the other methods. Although MI and MRMR show slight improvements over SARBO at later iterations, these gains occur only after substantially more evaluations, indicating that they require greater sampling effort for marginal benefit. These results are consistent with the ability of SISSO to explore large spaces of possibly nonlinear expressions, thus incorporating important nonlinear interactions when selecting relevant features.

To assess the generality of the performance trends observed with the GP-EI setup, we have also performed after-the-fact BO runs using RF as the surrogate model and UCB as the acquisition function (Fig. S2). Compared to GP-EI, it is observed that the RF-UCB-based BO campaigns show slightly faster convergence in early iterations. The high Δ*d*^C–O^_max_ values are identified at *N*_iter_ = 7 instead of 17 for SARBO. Nevertheless, the performance of BO with different FS methods remains the same: SARBO > MI > MRMR > RANDOM. These results suggest that SARBO’s adaptive feature selection provides benefits for BO experiments involving alternate choices surrogate models and acquisition functions.

**Fig. 5:**
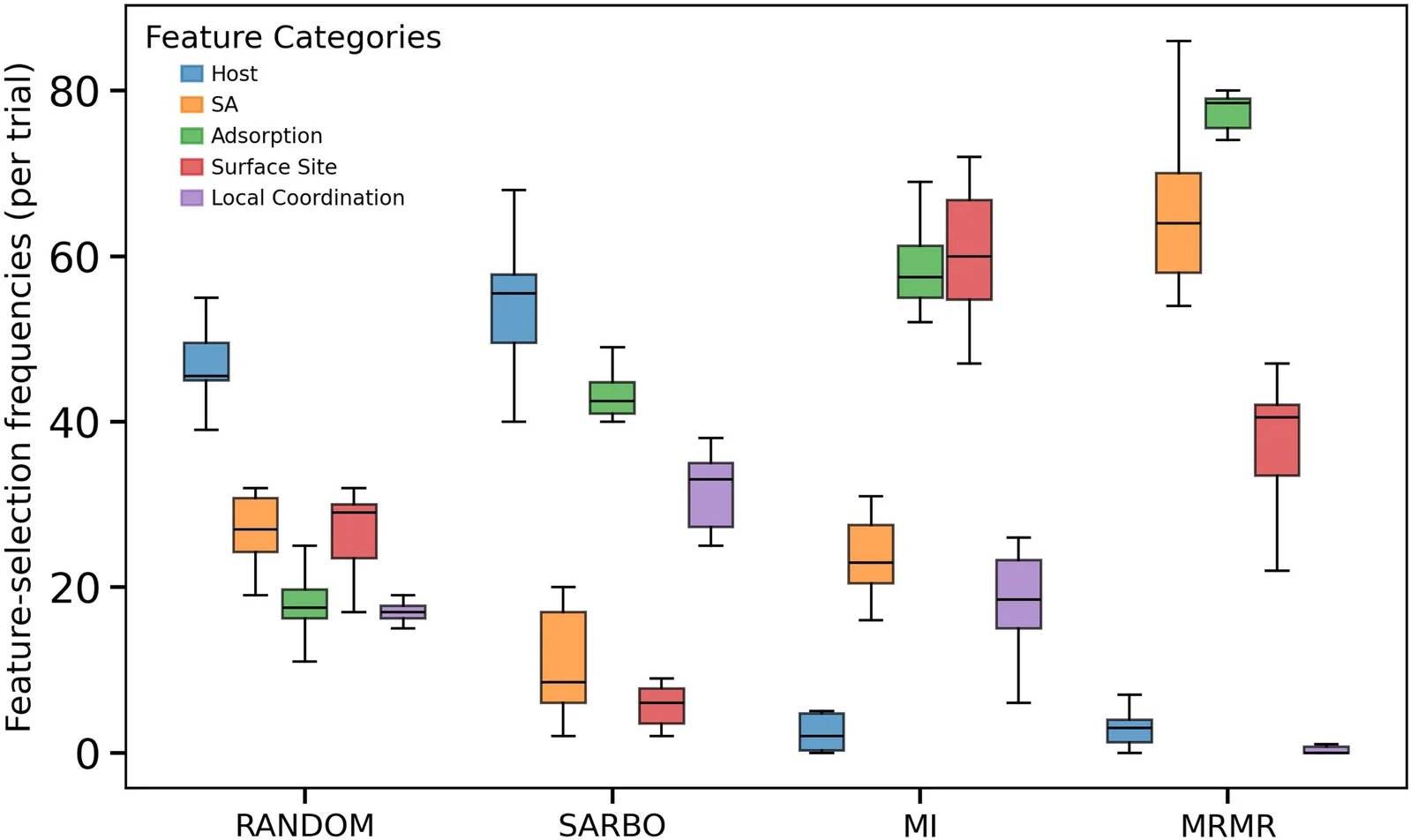
Distribution of feature category selection frequencies across 10 BO trials using GP-EI. For each category, boxplots display the distribution of cumulative selection counts per trial, with the central line indicating the median, box boundaries representing the interquartile range and whiskers showing the full data range.

To understand the origin of the difference in BO performance of different FS methods, we investigated which types of features were selected by each FS method. The five types of features contained in the dataset are host (8 features), dopant (7 features), CO_2_ adsorption (2 features), surface site (4 features), and local coordination environment (5 features) – see Methods section for details. We computed the total frequency of selection for each feature category per trial, summing over all 40 iterations shown in Fig. 4. The box plots in Fig. 5 show the distribution of these aggregated counts from the 10 trials. Distinct selection patterns emerge across different FS methods. The random feature selection exhibits the expected uniform distribution across categories, serving as an unbiased baseline. SARBO demonstrates a strong preference towards host- and adsorption-related features, followed by local-coordination related ones. MI primarily selects surface sites related features, whereas MRMR preferes SA-related features. All methods select adsorption-related features to some extent, consistent with their direct correlation to the target property Δ*d*^C–O^_max_. The distinctive feature selection pattern of SARBO suggests it identifies alternative physical relationships that may capture different aspects of the catalytic behavior. This unique feature selection pattern enables SARBO to effectively guide the optimization toward promising regions of the design space, as evidenced by its superior performance in identifying CO_2_-activating sites.

## 4 Conclusions

This work introduces the Sparse Adaptive Representation-based Bayesian Optimization (SARBO) framework, which applies the SISSO method for efficient feature selection. Crucially, the feature selection is performed at each iteration of the optimization. SARBO addresses the challenge of high dimensional feature spaces in BO, and it thus improves the efficiency of BO when the few, key features required to describe the materials property or function are not known beforehand. We demonstrate the superior optimization performance of SARBO compared to standard BO and popular feature selection methods for the identification of surface sites of single and dual-atom alloys capable of activating the CO_2_ molecule. We ascribe its superior performance to the ability to capture non-linear relationships and interaction between features that are often critical in governing target material properties but overlooked by widely used feature selection methods. Indeed, the features selected by SISSO take into account the different factors that contribute to the CO_2_ activation, such as the chemistry and surface structure of host, adsorption energetics and the effect of local coordination of the single-atom on the host surface. Furthermore, SARBO introduces a vital layer of interpretability to the typically black-box BO process. We anticipate that this work will enhance closed-loop frameworks by enabling more efficient exploration of vast design spaces, accelerating materials discovery efforts both for computational studies and for autonomous experimentation.

## Author contributions

Conceptualization, methodology, software: A. S. N.; writing: A. S. N., L. F.; supervision: M. S.

## Conflicts of interest

The authors declare no conflicts of interest.

## Supplementary Material

FD-267-D5FD00159E-s001

## Data Availability

The code base of SARBO, along with all the datasets and supporting scripts, is available at the GitLab repository https://gitlab.com/akhilsnair/SARBO. Supplementary information (SI) is available. See DOI: https://doi.org/10.1039/d5fd00159e.
