# Supplementary material for: Interpretable Bayesian optimization for catalyst discovery
Source: Faraday Discuss. 2026 Jan 27. Online ahead of print. doi: 10.1039/d5fd00159e (PMC13127247; doi:10.1039/d5fd00159e)
Supplement: FD-OLF-D5FD00159E-s001 [file FD-OLF-D5FD00159E-s001.pdf]

Supplementary Information (SI) for Faraday Discussions.
This journal is © The Royal Society of Chemistry 2026

# Supplementary Information: Interpretable Bayesian Optimization for Catalyst Discovery

Akhil S. Nair,[1,2] Lucas Foppa,[1] and Matthias Scheffler[1]

[1]The NOMAD Laboratory at the Fritz Haber Institute of the Max Planck Society, Faradayweg 4-6, D-14195 Berlin, Germany

[2]Institut für Chemie und Biochemie, Freie Universität Berlin, Arnimallee 22, 14195 Berlin, Germany

**Table S1:** List of features in the SAA dataset used in the study. The features are characterized based on whether they are related to the host, dopant single-atom, surface site, local coordination or adsorption.

| Type | Symbol | Unit | Description |
|---|---|---|---|
| **Host** | | | |
| | $PE_h$ | - | host Pauling electronegativity |
| | $IP_h$ | eV | host ionization potential |
| | $EA_h$ | eV | host electron affinity |
| | $r_{s-h}$ | Å | host s-orbital atomic radius |
| | $r_{p-h}$ | Å | host p-orbital atomic radius |
| | $r_{d-h}$ | Å | host d-orbital atomic radius |
| | $r_{val-h}$ | Å | host valence radius |
| | $bulk_{h-nnd}$ | Å | neighbor distance in host bulk |
| **Single-atom** | | | |
| | $PE_{SA}$ | - | SA Pauling electronegativity |
| | $IP_{SA}$ | eV | SA ionization potential |
| | $EA_{SA}$ | eV | SA electron affinity |
| | $r_{s-SA}$ | Å | SA s-orbital atomic radius |
| | $r_{p-SA}$ | Å | SA p-orbital atomic radius |
| | $r_{d-SA}$ | Å | SA d-orbital atomic radius |
| | $r_{val-SA}$ | Å | SA valence radius |
| **Surface site** | | | |
| | $PE_{site}$ | - | surface site PE[a] |
| | $IP_{site}$ | eV | surface site IP[a] |
| | $EA_{site}$ | eV | surface site EA[a] |
| | $site_{no}$ | # atoms | atoms in the surface site |
| **Local coordination** | | | |
| | $PE_{snn}$ | - | surface site and first neighbors PE[a] |
| | $IP_{snn}$ | eV | surface site and first neighbors IP[a] |
| | $EA_{snn}$ | eV | surface site and first neighbors EA[a] |
| | CN | # atoms | surface site coordination number |
| | gen-CN | # atoms | generalized CN |
| **Adsorption** | | | |
| | $E_{ads}$ | eV | Adsorption energy of $CO_2$ |
| | $O\text{-}C\text{-}O_{ang}$ | degree | O-C-O bond angle |

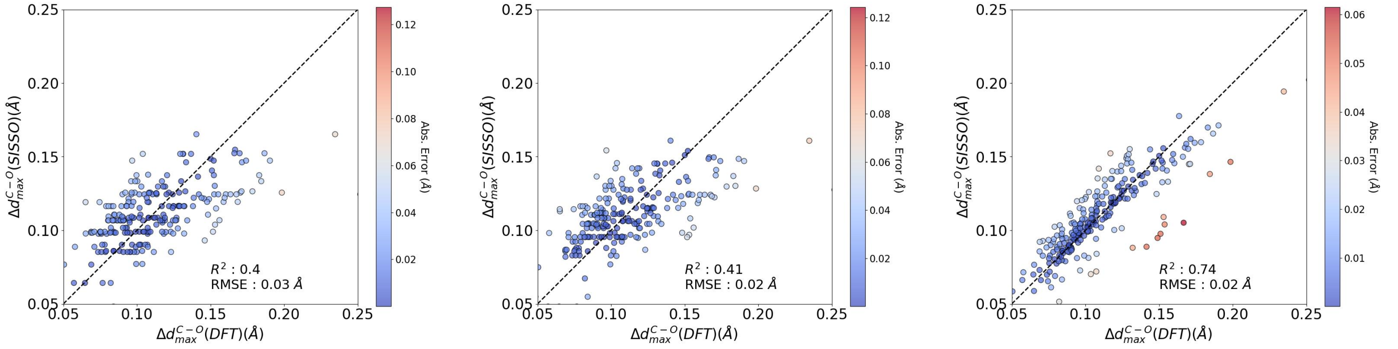


(a) (b) (c)

**Fig. S1**: Training performance of SISSO model (a) without using adsorption-related properties as features, (b) with the hi-SISSO approach adding the model and descriptors obtained from another SISSO model for adsorption energy prediction to the set of features and (c) including adsorption-related properties directly as features. The x- and y-axes of the parity plots show the true and predicted maximum C-O bond elongation ($\Delta d_{\rm max}^{\rm C-O}$) values, respectively.

**Table S2** : Features selected by different feature selection methods for predicting $\Delta d_{\rm max}^{\rm C-O}$. PE denotes Pauling electronegativity, IP ionization potential, EA electron affinity, rs/rp/rd/rval represent s-, p-, d-orbital and valence radii respectively, CN coordination number, and subscripts indicate: h (host), sa (single-atom), site (surface site), snn (surface site with first neighbors in the coordination environment). The features related to host, single-atom, site, adsorption and local coordination are indicated by cyan, orange, magenta, green and violet colours, respectively.

| **FS Method** | **Selected Features** |
|---|---|
| SISSO | CN, $\rm OCO_{ang}$, $\rm PE_{h}$, $\rm PE_{sa}$, $\rm gen_{CN}$, $\rm rs_{h}$, $\rm site_{no}$ |
| MI | $\rm OCO_{ang}$, $\rm PE_{site}$, $\rm rval_{sa}$, $\rm EA_{site}$, $\rm IP_{site}$, $\rm rd_{sa}$, $\rm rs_{sa}$ |
| RFE | $\rm E_{ads}$, $\rm OCO_{ang}$, $\rm EA_{sa}$, $\rm rd_{sa}$, $\rm IP_{snn}$, $\rm EA_{snn}$, $\rm gen_{CN}$ |
| MRMR | $\rm EA_{site}$, $\rm EA_{sa}$, $\rm PE_{sa}$, $\rm PE_{site}$, $\rm rd_{sa}$, $\rm E_{ads}$, $\rm OCO_{ang}$ |

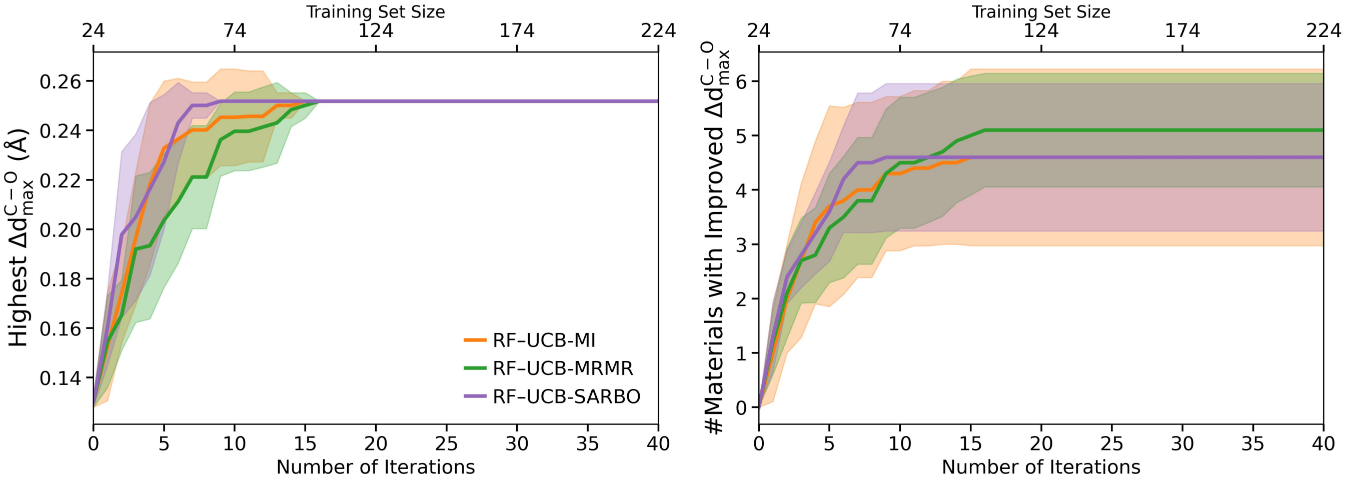


**Fig. S2.** Performance comparison between BO with different feature selection strategies using RF as the surrogate model and UCB as the acqusition function: (left) maximum $\Delta d_{\rm max}^{\rm C-O}$ evolution, (right) cumulative improvements in target property. Shaded regions indicate standard deviation across 10 independent trials.
